# Mitochondria‐targeted nanoparticles in treatment of neurodegenerative diseases

**DOI:** 10.1002/EXP.20210115

**Published:** 2021-12-28

**Authors:** Yue Zhang, Han Yang, Daohe Wei, Xinhui Zhang, Jian Wang, Xiaoli Wu, Jin Chang

**Affiliations:** ^1^ School of Life Sciences Tianjin University 92 Weijin Road, Nankai District Tianjin P. R. China; ^2^ School of Life and Health Science The Chinese University of Hong Kong Shenzhen P. R. China

**Keywords:** mitochondrial dysfunction, nanoparticles, neurodegenerative diseases, oxidative stress, target

## Abstract

Neurodegenerative diseases (NDs) are a class of heterogeneous diseases that includes Alzheimer's disease, Parkinson's disease, Huntington's disease, and amyotrophic lateral sclerosis. Mitochondria play an important role in oxidative balance and metabolic activity of neurons; therefore, mitochondrial dysfunction is associated with NDs and mitochondria are considered a potential treatment target for NDs. Several obstacles, including the blood‐brain barrier (BBB) and cell/mitochondrial membranes, reduce the efficiency of drug entry into the target lesions. Therefore, a variety of neuron mitochondrial targeting strategies has been developed. Among them, nanotechnology‐based treatments show especially promising results. Owing to their adjustable size, appropriate charge, and lipophilic surface, nanoparticles (NPs) are the ideal theranostic system for crossing the BBB and targeting the neuronal mitochondria. In this review, we discussed the role of dysfunctional mitochondria in ND pathogenesis as well as the physiological barriers to various treatment strategies. We also reviewed the use and advantages of various NPs (including organic, inorganic, and biological membrane‐coated NPs) for the treatment and diagnosis of NDs. Finally, we summarized the evidence and possible use for the promising role of NP‐based theranostic systems in the treatment of mitochondrial dysfunction‐related NDs.

## Graphical abstract

This review summarized the advantages and challenges of nanotechnology‐based mitochondrial‐targeted therapy for neurodegenerative diseases (NDs). In this review, relationships between mitochondrial dysfunction and NDs are discussed; the applications of various NPs in treatment and diagnosis of mitochondrial dysfunction related NDs are also introduced.

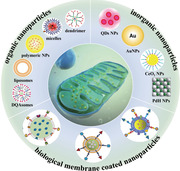

## 1 INTRODUCTION

Mitochondrion is one of the largest organelles in human cells, occupying approximately 25% of the cytoplasmic volume.^[^ ^1^ ^]^ It is an essential organelle for most eukaryotic cells, and plays a crucial role in cell survival/death. It is best known as the primary production site of the cellular adenosine triphosphate (ATP),^[^ ^2^ ^]^ and is responsible for energy distribution throughout the cell.^[^ ^3^ ^]^ In addition, mitochondria are involved in key cellular processes such as calcium homeostasis,^[^ ^4^, ^5^ ^]^ generation of reactive oxidation species (ROS),^[^ ^6^ ^]^ initiation of apoptosis,^[^ ^7^ ^]^ and release of metabolites to control cell fate and function.^[^ ^8^ ^]^ Hence, impaired mitochondrial function can radically alter cell and tissue homeostasis.

A growing number of studies have suggested that mitochondrial dysfunction occurs in healthy aging and diseases,^[^ ^9^ ^]^ especially neurodegenerative diseases (NDs).^[^ ^10^ ^]^ For instance, mitochondrial dysfunction induces the accumulation of phosphorylated tau (p‐tau) and Aβ in Alzheimer's disease (AD).^[^ ^11^, ^12^ ^]^ Moreover, most familial Parkinson's disease (PD) loci are directly related to the mitochondria.^[^ ^13^ ^]^ In brain samples obtained post‐mortem from PD patients, the mitochondrial respiratory chain was found to be dysfunctional.^[^ ^14^ ^]^ In addition, mitochondria isolated from cortical tissue obtained postmortem from Huntington's disease (HD) patients showed ultrastructural abnormalities.^[^ ^15^ ^]^ In postmortem analysis of spinal cord samples from amyotrophic lateral sclerosis (ALS) patients, reduced mitochondrial DNA (mtDNA) copy number and defective respiratory chain activity were found.^[^ ^16^ ^]^ Therefore, neuronal mitochondria may be a potential treatment target for NDs.

However, the target efficiency of most drugs for the mitochondria in brain parenchyma is quite limited, which affects the therapeutic effects in central nervous system (CNS) diseases. Typically, almost no macromolecular or small molecule drugs (>98%) are able to reach CNS^[^ ^17^, ^18^ ^]^ because of factors, including reduced drug diffusion through the blood‐brain barrier (BBB), lipophilic nature of drugs, and high negative potential of the mitochondria. These barriers limit the target efficiency of therapeutic drugs for brain and neuronal mitochondria. Hence, a high systemic dose is required to reach therapeutic levels within the brain, which leads to adverse effects.^[^ ^19^ ^]^

Nanotechnology‐based approach, defined as the use of dispersed solid particles of size 1–1000 nm, is a promising alternative strategy to treat NDs. Several nanoparticles (NPs) are approved for clinical use in the diagnosis or treatment of various diseases.^[^ ^20^, ^21^, ^22^, ^23^ ^]^ NPs are designed to allow them to successfully cross the BBB and target the cells or subcellular units.^[^ ^24^ ^]^ In addition, the increased surface area‐to‐volume ratio of NPs increases the rate of drug loading, thereby improving drug efficacy and safety. Moreover, NP surface modifications (e.g., the addition of peptides,^[^ ^25^ ^]^ biotin,^[^ ^26^ ^]^ or folic acid^[^ ^27^ ^]^) or conjunction with polyethylene glycol (PEG) enhances the ability of NPs to target lesions and prevent immune recognition.^[^ ^28^ ^]^ Notably, various routes of administration can be used to deliver NPs to the brain, including oral, intranasal, and parenteral routes.^[^ ^29^, ^30^ ^]^

**SCHEME 1:**
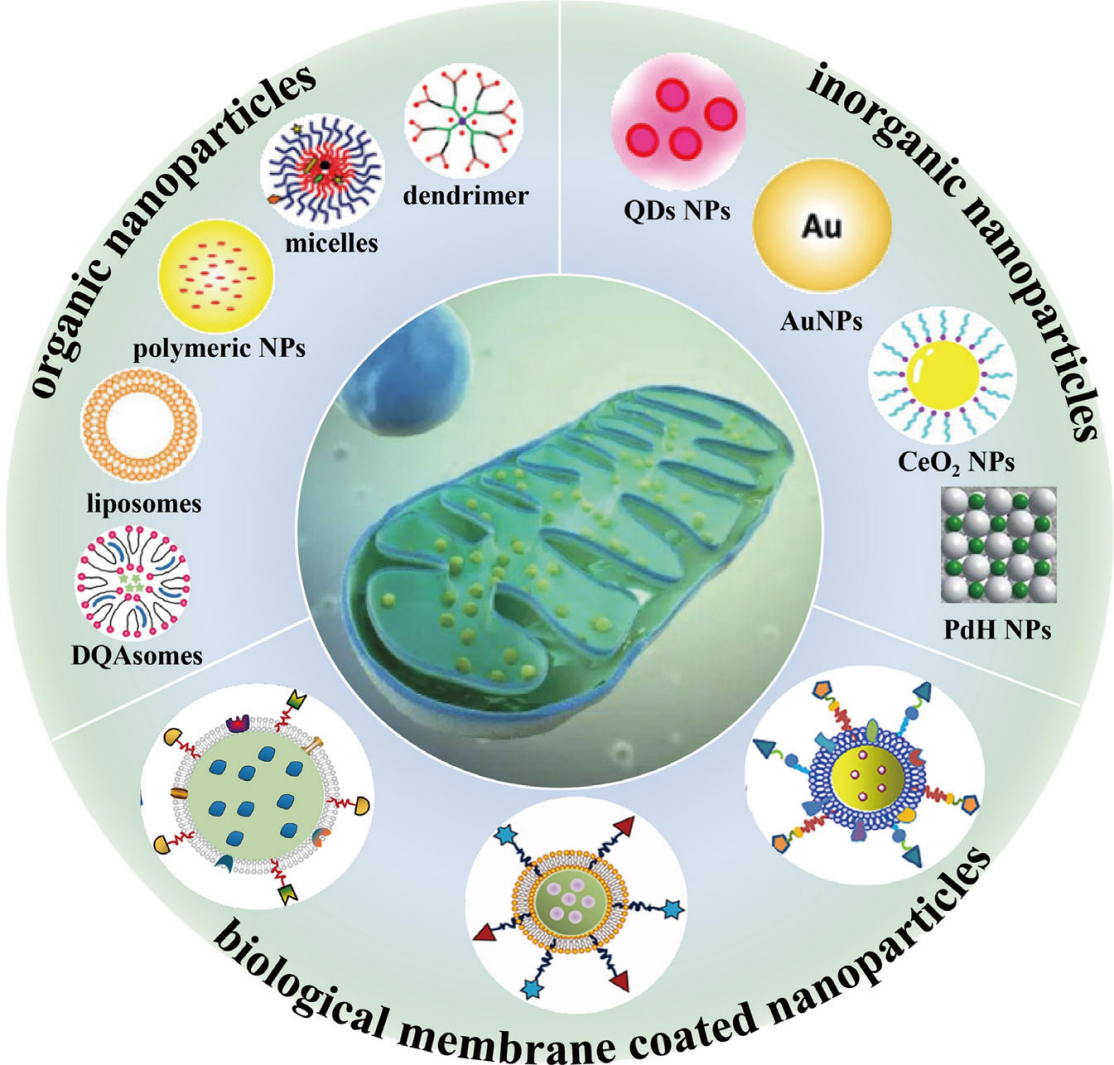
Schematic illustration of various nanoparticles‐based theranostic for mitochondrial dysfunction related NDs. Reproduced with permission. Copyright from 2020 Wiley‐VCH GmbH;^[^ ^31^ ^]^ 2016 American Chemical Society;^[^ ^32^ ^]^ 2019 Elsevier Ltd. All rights reserved;^[^ ^33^ ^]^ 2020 The Authors. Publishing services by Elsevier B.V. on behalf of KeAi Communications Co., Ltd;^[^ ^34^ ^]^ 2020 The Author(s). Published by Informa UK Limited, trading as Taylor & Francis Group;^[^ ^35^ ^]^ 2020 Acta Materialia Inc. Published by Elsevier Ltd. All rights reserved.^[^ ^36^ ^]^

This review summarized the advantages and challenges of nanotechnology‐based mitochondrial‐targeted therapy for NDs. First, mitochondrial structure and function are discussed, followed by a discussion of the relationships between mitochondrial dysfunction and pathogenesis of four NDs. Second, the therapeutic barriers and treatment strategies are discussed. Third, nanotechnology‐based strategies for mitochondrial‐targeted treatment (Scheme 1) using organic, inorganic, and biological membrane‐coated NPs are discussed. Various NPs are designed and used for the diagnosis and treatment of NDs. The final sections describe the prospects of use of mitochondrial‐targeted nanotherapy for NDs. Further studies are required to develop optimal nanotechnology‐based treatments for mitochondrial dysfunction‐related NDs.

## 2 THERAPEUTIC BARRIERS AND TREATMENT STRATEGIES RELATED TO NEURONAL MITOCHONDRIAL DYSFUNCTION

### 2.1 Mitochondrial structure and function

Mitochondria are closed sac‐like structures composed of two membrane layers (outer mitochondrial membrane [OMM] and inner mitochondrial membrane [IMM]), which differ in chemical and structural composition. The OMM contains equal proportions of proteins and lipids, and it can be penetrated by molecules with size <6 kDa.^[^ ^37^ ^]^ IMM is a highly specialized unit membrane that folds inward to form a ridge, thereby increasing its surface area. IMM has a low permeability and proteins account for 76% of its total weight. Many substances (such as H^+^, ATP, and pyruvate) require carriers to penetrate the IMM. The intermembrane space lies between the OMM and IMM. The chamber enclosed within the IMM is the mitochondrial matrix, which contains various enzymes, mtDNA, ribosomes, and RNA.^[^ ^38^ ^]^ These characteristics may be used to design targeted strategies to the mitochondria.

**FIGURE 1:**
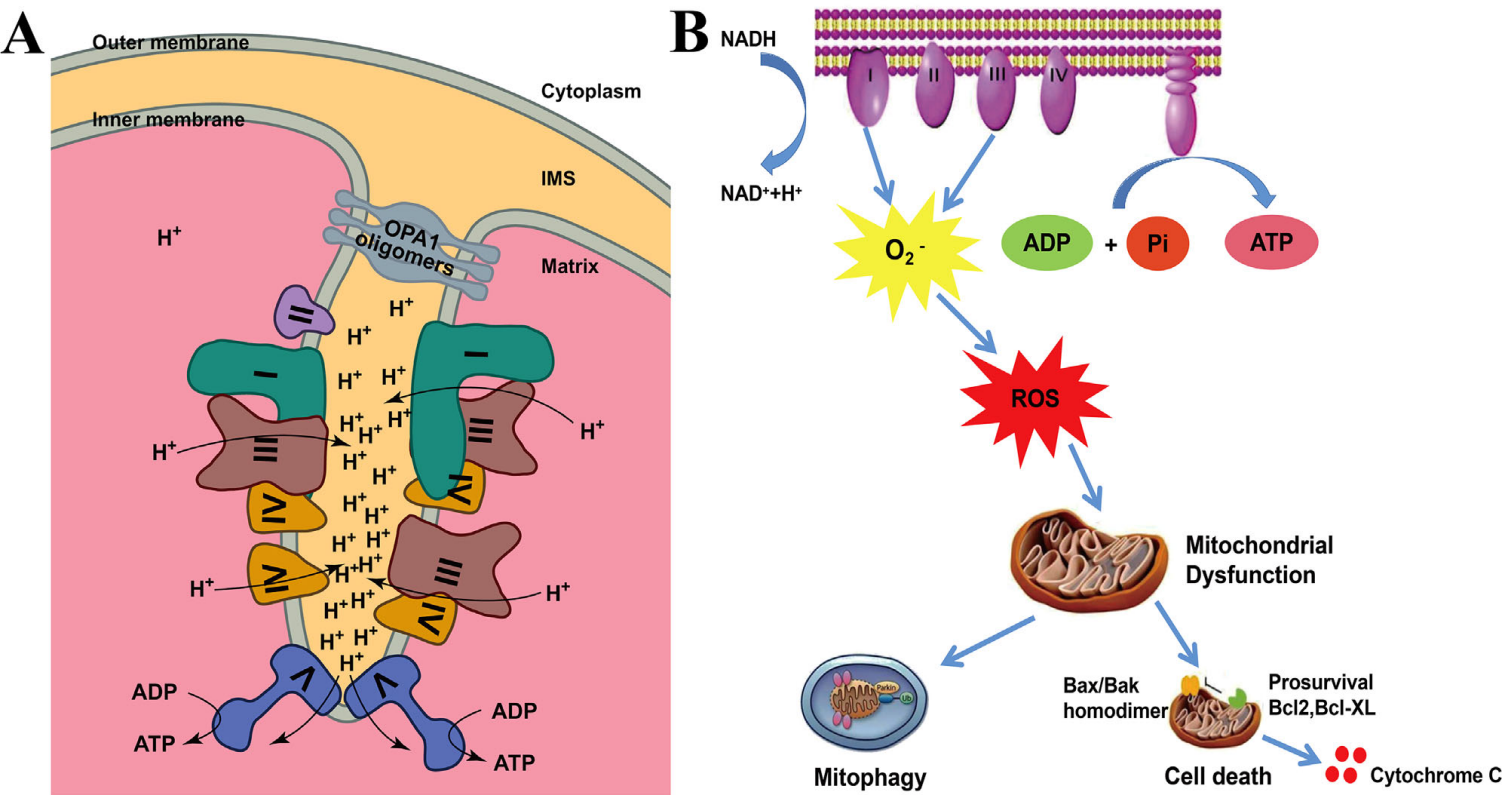
Schematic illustration of mitochondrial ETC and mitochondrial dysfunction. (A) Simple cartoon model of ETC on the IMM. Reproduced with permission.^[^ ^43^ ^]^ Copyright © 2017 Chang Gung University. Publishing services by Elsevier B.V. (B) Electron leakage from ETC induces superoxide ions, generates excessive ROS, and causes mitochondria damage as well as subsequent events. Reproduced with permission.^[^ ^42^ ^]^ Copyright © 2019 Wiley Periodicals, Inc.

The main function of mitochondria is to supply ATP and oxygen to the cell, which involves the transport of electrons along the electron transport chain (ETC).^[^ ^39^ ^]^ The ETC contains five proteins on the IMM: complexes I–V (Figure 1A). The complexes I, III, and IV act as the proton pumps in electron transfer and transfer H^+^ from the intermembrane space to the matrix. Therefore, a proton gradient is created (negative internal membrane potential, ΔΨm), which produces a strong negative potential on the IMM of about −160 to −180 mV^[^ ^40^ ^]^ and affects drug entry into the mitochondria. Interestingly, about 0.4–4% of the electrons passing through the ETC will not be fully restored, leading to the production of primary ROS‐superoxide anion (•O^2−^) production. Excessive generation of superoxide anions interacts with many other compounds to generate secondary ROS and induce oxidative damage.^[^ ^41^ ^]^ High concentration and prolonged exposure to ROS causes mitochondrial damage. When the rate of mitophagy cannot catch up with the production of dysfunctional mitochondria, the apoptosis signal pathway is activated (Figure 1B).^[^ ^41^, ^42^ ^]^

### 2.2 NDs and mitochondrial dysfunction

NDs, including AD, PD, HD, and ALS, are a class of heterogeneous diseases characterized by progressive and selective loss of neurons.^[^ ^10^ ^]^ Despite the heterogeneity, mitochondrial dysfunction plays an important role in ND pathogenesis.^[^ ^10^ ^]^

Neurons are metabolically active and rely on mitochondria for energy. As a highly oxygen‐consuming organ, the brain requires nearly 20% of the total oxygen in the whole body.^[^ ^44^ ^]^ However, it has a weak antioxidant defense system. Owing to the high energy requirements and high rate of ATP production/consumption in the brain, a large number of ROS by‐products are generated. ROS overproduction or antioxidant system dysregulation impairs normal mitochondrial function,^[^ ^41^ ^]^ leading to inflammation, apoptosis, memory loss, and brain/neuropathology damage.^[^ ^45^ ^]^ Because their membrane is rich in polyunsaturated fatty acids, neurons are very sensitive to ROS and brain is vulnerable to mitochondrial related oxidative damage.^[^ ^41^ ^]^ Furthermore, the mtDNA encodes for a small number of genes associated with complexes I–V and is not protected by histones. Therefore, the mutation rate of mtDNA is several times higher than that of nuclear DNA.^[^ ^46^ ^]^ Age‐related deletions and point mutations lead to mitochondrial dysfunction and induce the development of NDs.^[^ ^47^ ^]^ ROS production and accumulation of mtDNA damage/mutations induce mitochondrial dysfunction, which causes ND progression.

Several previous studies have reported similar results. Neuronal mitochondrial dysfunction induced by persistent ROS production accelerates the pathological process underlying AD, such as increased Aβ aggregation and production of neurofibrillary tangles.^[^ ^48^, ^49^ ^]^ Furthermore, mitochondrial dysfunction induces DNA damage and is involved in PD pathogenesis.^[^ ^50^ ^]^ In addition, mitochondrial dysfunction due to high ROS levels induces protein misfolding in HD, leads to aggregation of inclusion bodies in the axons and dendrites of neurons, and inhibits neurotransmitter delivery.^[^ ^51^ ^]^ Moreover, in vivo studies of ALS confirm that mitochondrial dysfunction and deficient oxidative phosphorylation occur before the disease onset.^[^ ^44^, ^52^ ^]^ Additionally, mitochondrial dysfunction results in activation of caspase‐3 and neuronal apoptosis in NDs.^[^ ^53^ ^]^ The relationships between the aforementioned NDs and mitochondrial dysfunction are summarized in Table 1.

**TABLE 1:** Relationship of four NDs and mitochondrial dysfunction

**Diseases**	**Relationship with mitochondrial dysfunction**	**Ref**.
AD	✓ Altered mitochondrial morphology, including broken cristae, swelling, and random distribution. ✓ P‐tau blocks mitochondrial transport and leads to oxidative stress and energy deprivation. ✓ Reduced oxygen metabolism and defective activity of mitochondrial enzymes. ✓ A reduction of mtDNA content in mitochondria of neurons and cerebrospinal fluid.	^[^ ^11^, ^54^, ^55^ ^]^
PD	✓ Mitochondrial morphology change: reduced cristae and swelling. ✓ Defective mitochondrial respiratory chain (in particular respiratory chain complex I) and mtDNA in midbrain substantia nigra. ✓ Pathogenic gene mutations are directly related to mitochondrial dysfunction. ✓ Mutations, deletions, and rearrangements of mtDNA in neuronal mitochondria.	^[^ ^13^, ^56^, ^57^ ^]^
HD	✓ Ultrastructural abnormalities of HD cortical mitochondria. ✓ Interaction of OMM and mutant huntingtin triggers abnormal mitochondrial morphology. ✓ Defective complexes II, III, and IV in striatum. ✓ Impaired complexes I/III improve mitochondrial generated O ^2 •−^ level. ✓ Decreased ΔΨm.	^[^ ^10^, ^58^, ^59^, ^60^ ^]^
ALS	✓ Many ALS genes are directly related to mitochondrial function (such as superoxide dismutase 1 gene). ✓ Enriched mitochondrial clusters in lumbar spinal cord. ✓ Deletions of mtDNA in neuronal mitochondria. ✓ Neuronal mitochondrial swelling.	^[^ ^16^, ^61^, ^62^ ^]^

Abbreviations: AD: Alzheimer's disease; mtDNA: mitochondrial DNA; ALS: amyotrophic lateral sclerosis; PD: Parkinson's disease; HD: Huntington's disease; OMM: outer mitochondrial membrane.

### 2.3 Therapeutic barriers and treatment strategies of NDs

**FIGURE 2:**
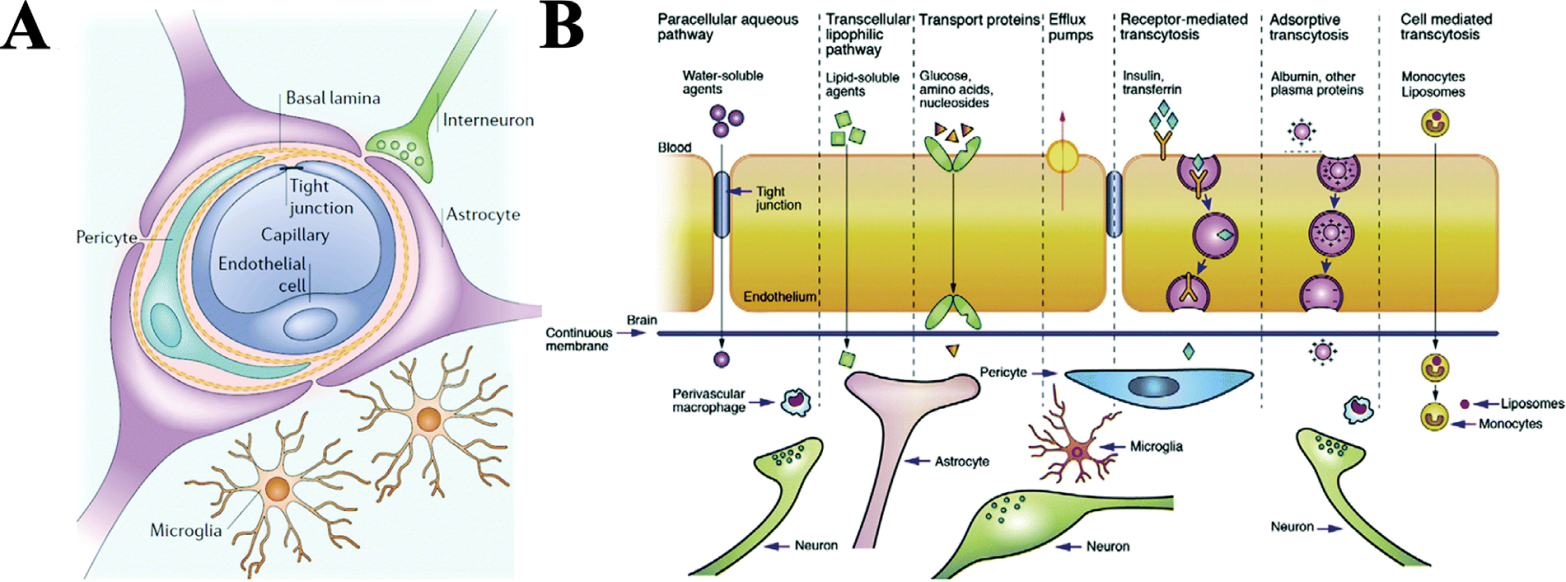
Structure of BBB and potential routes of molecules transportation. (A) Schematic illustration about cellular composition of the BBB. (B) Schematic illustration of different molecules transport pathways of BBB. Reproduced with permission.^[^ ^73^ ^]^ Copyright © The Royal Society of Chemistry 2019

Drugs need to overcome a number of biological barriers, including the BBB, cell membrane, and mitochondrial membrane, to reach the neuronal mitochondria in vivo (Table 2).^[^ ^31^, ^37^ ^]^ The primary obstacle of drug delivery to neuronal mitochondria is the BBB.^[^ ^63^ ^]^ The BBB is a highly selective interface between brain parenchyma and blood,^[^ ^64^ ^]^ formed by different cell types and possessing unique structural features. The BBB primarily consists of cerebral capillary endothelial cells, which overlap to form tight junctions that effectively prevent macromolecules from passing through the endothelial cell junction. In addition, pericytes partially enclose and share a common basal lamina with endothelial cells, whereas perivascular endfoot of astrocytes surround about 85% of the cerebral capillaries to connect them to the neurons (Figure 2A).^[^ ^65^, ^66^ ^]^ Therefore, most drugs entry are excluded by the BBB.^[^ ^17^ ^]^ Recently, efforts have been made to overcome the BBB. Invasive methods of administration include intrathecal drug delivery.^[^ ^67^ ^]^ The noninvasive approaches include chemically modifying the drugs,^[^ ^68^ ^]^ using viruses/exosomes as vectors for CNS drug delivery,^[^ ^69^, ^70^ ^]^ intranasal drug delivery to bypass the BBB,^[^ ^71^ ^]^ transport systems for drug delivery (Figure 2B),^[^ ^17^ ^]^ destruction of the BBB by ultrasound or radiotherapy,^[^ ^72^ ^]^ and nanotechnology‐based strategies.

**TABLE 2:** Properties of biological barriers in targeting neuron mitochondria and potential ways to cross them

**Barriers**	**Properties**	**Ways to cross the barriers**	**Ref**.
BBB	✓ Cerebral capillary endothelial cells overlap to form tight junctions ✓ Pericytes partially enclose the endothelial cells ✓ Endfoot processes of astrocytes surround about 85% of cerebral capillaries	✓ Intrathecal administration ✓ Chemical modification of drugs ✓ Use of viruses/exosomes as vectors for CNS delivery ✓ Intranasal delivery to bypass the BBB ✓ Transport mechanism mediated drug delivery on the BBB ✓ Destruction of the BBB by ultrasound or radiotherapy ✓ Nanotechnology‐based strategies	^[^ ^67^, ^68^, ^69^, ^70^, ^71^, ^72^ ^]^
Cell membrane	✓ −70 mV potential difference ✓ Lipophilic membrane ✓ Selective permeability	✓ Positively charged ✓ Lipophilic molecules (<500 mw) ✓ Receptor ligand modification	^[^ ^29^, ^74^ ^]^
OMM	✓ Lipophilic barrier	✓ Membrane potential‐driven targeting ✓ Affinity‐driven targeting ✓ Mitochondrial transport systems driven targeting ✓ Nanotechnology‐based targeting	^[^ ^24^, ^31^, ^37^, ^76^ ^]^
IMM	✓ Rich in cardiolipin, higher protein‐to‐lipid ratio (3:1) ✓ Strong negative membrane potential (ΔΨm) of approximately −160 to −180 mV		

Abbreviations: BBB: blood‐brain barrier; OMM: outer mitochondrial membrane; IMM: inner mitochondrial membrane.

The other barrier to drug delivery is the cell membrane, which is a lipophilic membrane with selective permeability and −70 mV potential difference. Therefore, lipophilic and small molecular weight (mw) drugs can selectively penetrate the cell membrane.^[^ ^74^ ^]^ Other drugs can enter cells through macro‐pinocytosis, caveolae, and clathrin pathways.^[^ ^29^ ^]^

Notably, owing to the highly negative potential, lipophilic nature, and impermeability of mitochondria, it is difficult to selectively target drugs to the mitochondria in vivo.^[^ ^37^ ^]^ Drug delivery into the mitochondria must overcome two layers of biofilms: OMM and IMM. There is no membrane potential on OMM and lipophilic particles with a molecular weight < 5000 mw can freely penetrate through it. The IMM is a highly specialized unit membrane, with low permeability, which is rich in cardiolipin and has a higher protein‐to‐lipid ratio (3:1).^[^ ^74^ ^]^ Small lipophilic molecules (<500 mw) can pass through the IMM, whereas many other substances (such as H^+^, ATP, and pyruvate) require carriers for crossing the IMM. Interestingly, ΔΨm makes IMM resistant to anionic molecules.^[^ ^39^ ^]^ These mitochondrial characteristics inhibit drug delivery into the mitochondria for the treatment of NDs. Therefore, strategies are urgently needed to overcome these barriers.

Currently, mitochondrial targeting can be achieved using the following strategies: (1) Membrane potential‐driven targeting: triphenylphosphonium (TPP), the most widely studied mitochondrial targeting molecule, is a mitochondrial homing moiety with both lipophilic and hydrophilic properties.^[^ ^75^ ^]^ (2) Affinity driven targeting: short bacitracin S (GS) can destroy bacterial membranes. Due to the similar structures of mitochondria and bacteria, the GS derivative hemi‐GS has a high aggregation efficiency in the mitochondria.^[^ ^76^ ^]^ In addition, Szeto‐Schiller tetra‐peptide (SS peptide) is another mitochondrial targeting peptide that has a positive charge and is lipophilic.^[^ ^76^ ^]^ (3) Targeting driven by mitochondrial transport systems: more than 99% of mitochondrial proteins are synthesized in the cytoplasm and transported by the mitochondrial transport systems. Therefore, the N‐terminal mitochondrial‐targeted signal peptide is designed for the import of the peptide sequence into the mitochondria.^[^ ^77^ ^]^ (4) Nanotechnology‐based targeting: based on the mitochondrial characteristics, NPs are developed for mitochondrial targeting. Interestingly, nanotechnology‐based targeting shows superiority to the other strategies and combines most of the advantages of other strategies.

## 3 MITOCHONDRIAL‐TARGETED NPS FOR THE TREATMENT OF NDS

NPs have various advantages for the treatment of NDs.^[^ ^78^ ^]^ NPs have an adjustable size, appropriate charge, and lipophilic surface.^[^ ^24^ ^]^ The increased surface area to volume ratio of NPs improves the rate of drug loading. Thus, low doses of drugs are used in vivo with high efficacy and safety.^[^ ^79^ ^]^ NPs can be functionalized with PEG or targeted residues (e.g., peptides,^[^ ^25^ ^]^ biotin,^[^ ^26^ ^]^ and folic acid^[^ ^27^ ^]^) for increasing the duration of circulation^[^ ^28^ ^]^ or CNS targeting, respectively.^[^ ^80^ ^]^ They provide sustained and targeted drug delivery systems for the dysfunctional mitochondria in NDs.^[^ ^81^ ^]^ Here, we present different types of NPs that can be used to treat mitochondrial dysfunction‐related NDs.

### 3.1 Organic NPs

#### 3.1.1 Liposome‐based NPs

Liposomes are spherical nanosystems composed of hydrophobic cavities, surrounded by bilayer of natural amphiphilic lipids. The natural and dual nature of liposomes makes it superior to be captured by mitochondria for the delivery of both hydrophilic and hydrophobic substances.^[^ ^82^ ^]^ Currently, more than 20 formulations are being studied in clinical trials or have been approved for clinical use.^[^ ^83^ ^]^

**FIGURE 3:**
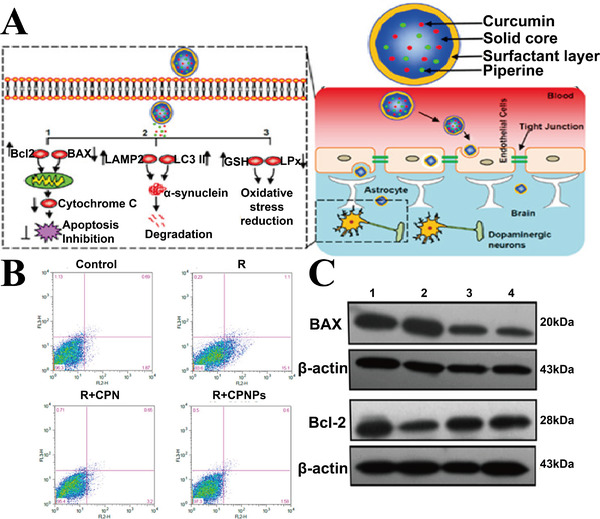
(A) Design and function of CP NPs in PD mouse model. (B) Apoptosis percentage of PC12 cells, in R (rotenone), CPN (free curcumin + piperine), or CP NPs treatment groups. (C) Expression of Bcl2 and BAX in different treatment groups. Group 1: control; group 2: R; group 3: R + CPN; group 4: R + CP NPs. Reproduced with permission.^[^ ^88^ ^]^ Copyright © 2016 American Chemical Society

In view of their biocompatibility and non‐toxic characteristics, lipid‐based formulations, such as liposomes, lipid NPs (nanostructured lipid carriers,^[^ ^84^, ^85^ ^]^ and solid lipid NPs (SLN),^[^ ^86^, ^87^ ^]^ have great potential for targeting the CNS and mitochondria. Coated with the surfactants F68 and vitamin E‐D‐α‐tocopherol poly(ethylene glycol) 1000 succinate (vitamin E‐TPGS), glyceryl‐monooleate self‐assembled lipid NPs, encapsulated with curcumin and piperine (CP NPs), are used for the treatment of PD (Figure 3A). CP NP treatment leads to significant reduction in the apoptosis ratio (Figure 3B) and substantial increase in the Bcl‐2/BAX ratio (anti‐apoptotic protein/pro‐apoptotic protein) (Figure 3C) compared to the rotenone‐treated PC12 cells.^[^ ^88^ ^]^

Furthermore, the effectiveness of SLN was evaluated. SLN encapsulated with thymoquinone (TQ‐SLNs) have been used for the treatment of HD rat model. Excessive hydroxyl radical production in the mitochondria leads to abnormal succinate dehydrogenase (SDH) activity. TQ‐SLNs increase the in vivo bio‐availability of free TQ by fivefold. Compared to the control group, SDH activity in rat striatum is increased by 20%, and the degeneration of mitochondria is significantly reduced, 14 days after TQ‐SLN treatment.^[^ ^87^ ^]^ Similarly, Sandhir et al. develop curcumin‐encapsulated SLNs (C‐SLNs), which improved the ability of curcumin to cross the BBB by 11.93‐fold. C‐SLN‐treated rats showed significant reduction in mitochondrial swelling (11.20%) as compared to the HD group (22.06%). Meanwhile, C‐SLN reduced the malonaldehyde (MDA) levels (31.23%) and ROS production (33.98%), and increased the locomotor activity (20.31%), thereby ameliorating the symptoms of HD.^[^ ^89^ ^]^

Additionally, TPP‐conjugated liposomes enhance the targeting capability, bioavailability, and CNS concentration of drugs.^[^ ^90^ ^]^ Boddapati et al. combined TPP with stearyl residues to create stearyl triphenyl phosphorus (STPP), which can be incorporated into the lipid bilayers. Mitochondria‐targeted STPP‐conjugated group showed higher co‐localization in the mitochondria compared to the non‐target group (Pearson coefficient = 0.39 ± 0.219), suggesting that STPP modifies the TPP residues on liposome NPs.^[^ ^91^, ^92^ ^]^

#### 3.1.2 Polymeric NPs

Owing to the ease of manufacture and flexible surface modification,^[^ ^93^ ^]^ high molecular polymers are widely used for altering hydrophobic drug pharmacokinetics and protecting drugs against environmental deactivation/degradation.^[^ ^94^, ^95^, ^96^ ^]^ Polymeric NPs are composed of different substances, including polysaccharides (such as chitosan,^[^ ^97^ ^]^ sodium alginate,^[^ ^98^ ^]^ and human serum albumin^[^ ^29^ ^]^), and synthetic polymers (such as poly[lactide‐co‐glycolide] [PLG],^[^ ^99^ ^]^ poly[lactic acid] [PLA],^[^ ^100^ ^]^ and poly[lactic‐co‐glycolic acid] [PLGA]^[^ ^101^ ^]^). Among them, PLGA NPs are approved for pharmaceutical use.^[^ ^102^ ^]^

Dequalinium (DQA) is an amphiphilic compound with two symmetrical cationic charge centers. DQA self‐assembles to positively charged liposome‐like structures called DQAsomes to penetrate the mitochondria. It is commonly used as a gene delivery system (DQAsome‐DNA complex).^[^ ^103^ ^]^ A green fluorescent protein (GFP) gene transfection system based on DQAsome has been synthesized, which can deliver GFP genes to the mitochondria of RAW264.7 cell line, albeit with low efficiency (1%–5%). Similar transfection efficiency has been found in other cell lines.^[^ ^104^ ^]^ DQAsomes loaded with curcumin significantly enhance the water solubility and antioxidant activity of drugs, and show good mitochondrial targeting ability in vitro.^[^ ^105^ ^]^

Unlike DQAsome, other polymeric NPs require modification of the targeted groups^[^ ^93^ ^]^ to improve mitochondrial targeting ability. Conjugated with TPP and fluorescein isothiocyanate (FITC), acetylated generation 5 poly(amidoamine) (PAMAM) dendrimer NPs (G(5)‐D‐Ac‐TPP NPs) can effectively target the mitochondria.^[^ ^106^ ^]^ Furthermore, by changing the ratio of lactic acid to glycolic acid (LA:GA) in PLGA, NPs eliminate the 1‐methyl‐4‐phenylpyridinium ion, a mitochondrial parkinsonian neurotoxin, and alleviates some PD‐related changes, such as increased ROS production, complex I inhibition, and BAX activation.^[^^]^

**FIGURE 4:**
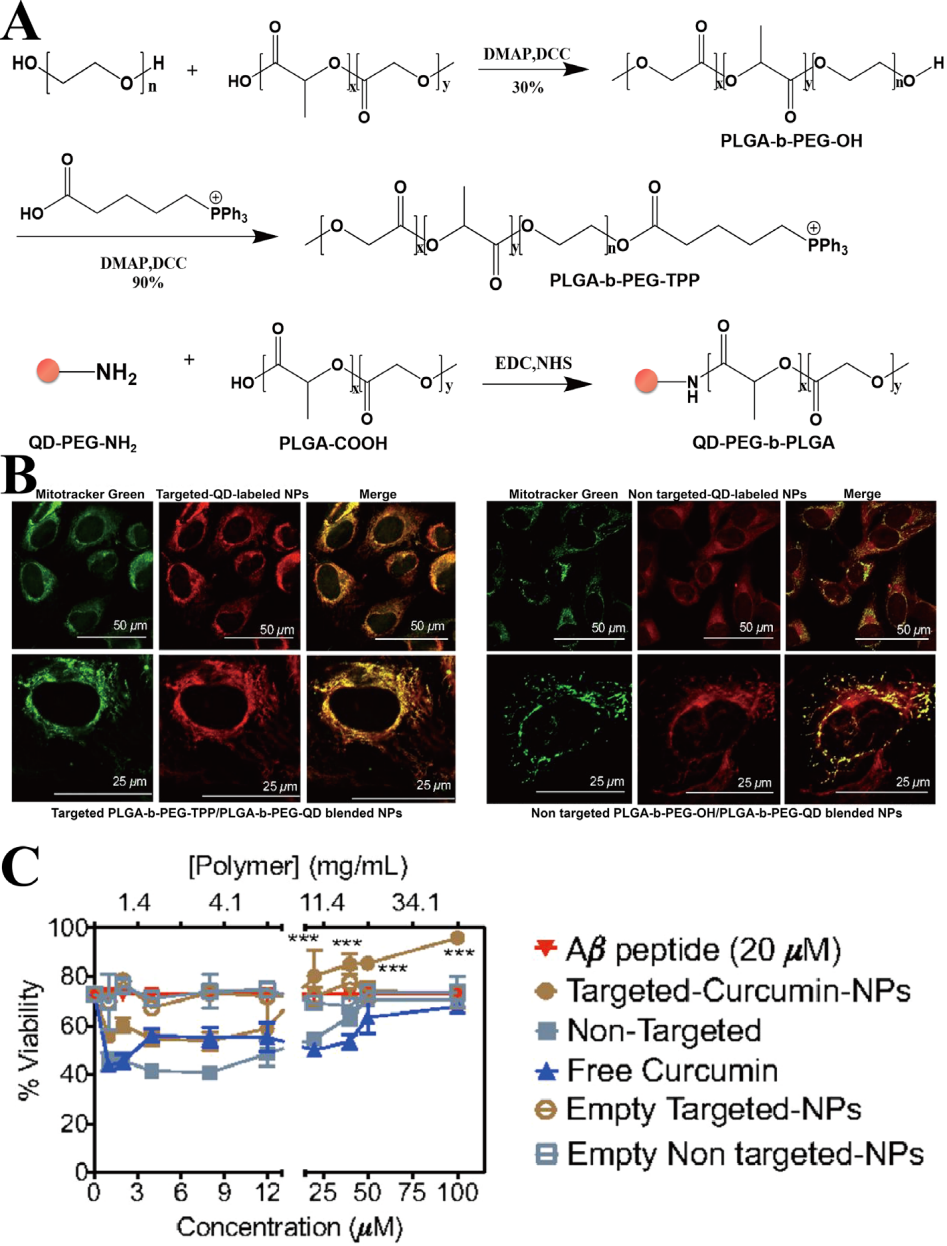
(A) Synthesis of PLGA‐b‐PEG‐QD polymer, PLGA‐b‐PEG‐TPP polymer, and PLGA‐b‐PEG‐OH polymer. (B) Co‐localization of targeted (left side, red) and non‐targeted NPs (right side, red) with MitoTracker (mitochondrial marker, green) (C) Survival rate of Aβ‐damaged neuron cells treated with free curcumin, nontargeted NPs loaded with curcumin, and targeted NPs loaded with curcumin. ***p < 0.001. Reproduced with permission.^[^ ^107^ ^]^ Copyright © 2012 National Academy of Sciences of the United States of America (NAS)

Micelles, composed of amphiphilic diblock or triblock copolymers, are smaller in size and have higher structural solubility than liposomes.^[^ ^17^ ^]^ Two kinds of polymer copolymers are synthesized: PLGA‐*b*‐PEG‐OH, with a single terminal TPP cation (PLGA‐*b*‐PEG‐TPP); and a powerful intracellular tracker quantum dot (QD) conjugated with PLGA‐*b*‐PEG (PLGA‐b‐PEG‐QD) (Figure 4A), to construct non‐target NPs (PLGA‐b‐PEG‐OH+PLGA‐b‐PEG‐QD) and target NPs (PLGA‐b‐PEG‐TPP+ PLGA‐b‐PEG‐QD). Results showed that the colocalization coefficient of target NPs (red) in mitochondria (green) is significantly higher than non‐target NPs group (Figure 4B). When curcumin is encapsulated, it can be used in the treatment of AD. Survival rate of Aβ‐induced neuron cells is higher in targeted NPs compared to other groups (Figure 4C) in vitro.^[^ ^107^ ^]^ Similarly, PLGA‐b‐PEG‐TPP polymer‐based NPs loaded with coenzyme Q_10_ (CoQ_10_) can effectively attenuate mitochondrial ROS production and increase cell membrane potential.^[^ ^108^ ^]^

**FIGURE 5:**
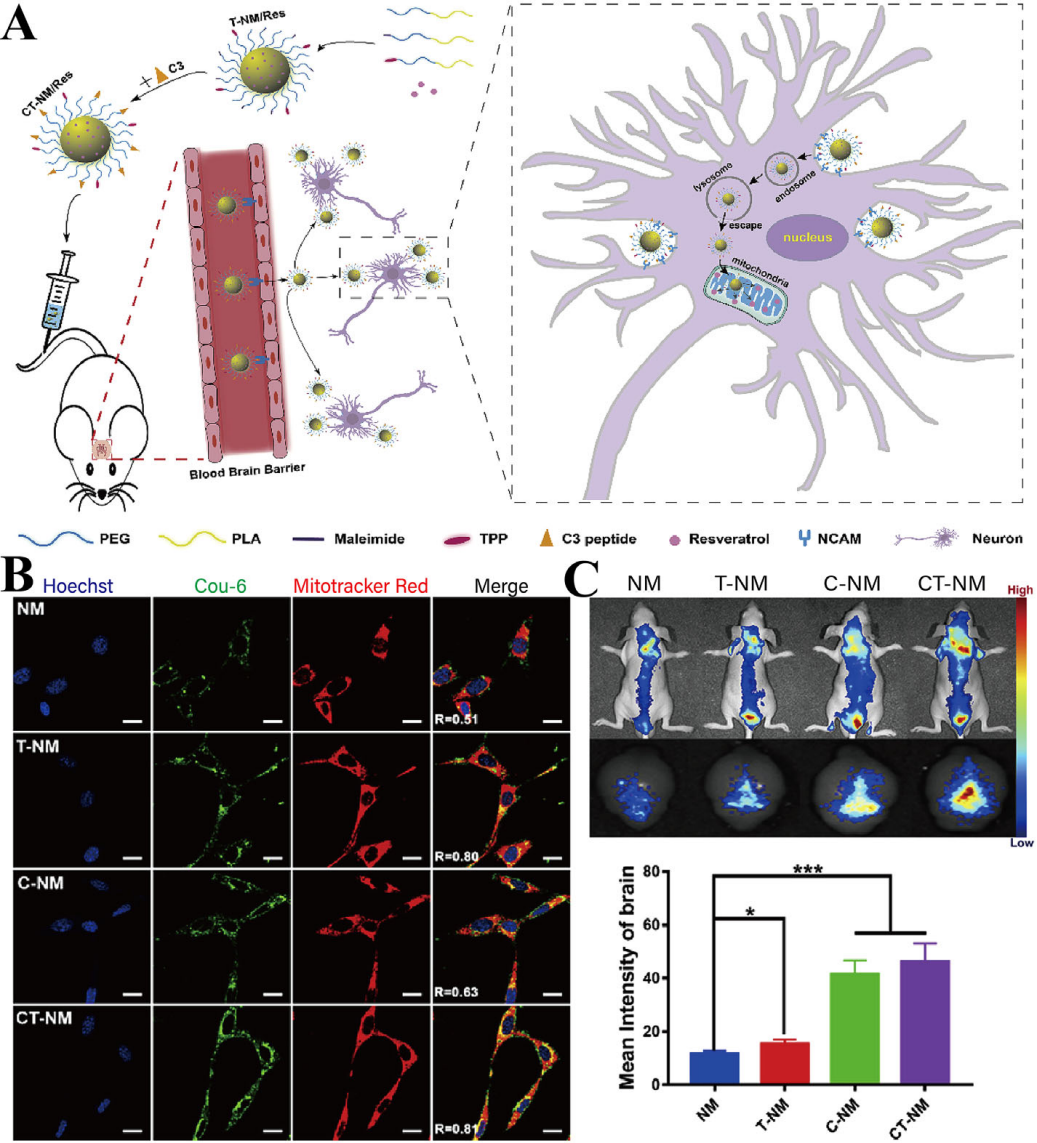
(A) Schematic illustration of the targeting ability and therapeutic function of micelles. (B) Co‐localization imaging of different Cou‐6‐loaded NPs group (green) with mitochondria (red) and nuclei of Neuro‐2a cells (blue) (scale bar = 10 μm). R denotes the overlap ratio. (C) Representative in vivo imaging (left), and statistics data (right) of brain mean fluorescence intensity 1 h post injection with different DiR‐loaded NPs. *p < 0.05, ***p < 0.001. Reproduced with permission.^[^ ^109^ ^]^ Copyright © 2020 Elsevier Ltd. All rights reserved

Yang et al. studied the in vivo therapeutic effects of micelles. Conjugated with mitochondrial‐targeting molecules TPP and neural cell adhesion molecule mimetic peptide C3, PEG‐PLA micelles (CT‐NM) were prepared to target neurons and mitochondria concurrently to treat AD (Figure 5A). CT‐NM loaded with Cou‐6 (green) accumulated in the cells and co‐localized with mitochondria (red) in merged images (co‐localization coefficient *R* = 0.81) (Figure 5B), indicating that CT‐NM has superior neuronal mitochondrial targeting ability compared with NM (PEG‐PLA micelles), T‐NM (micelles modified with TPP), and C‐NM (micelles modified with C3) groups. Compared with Aβ_25‐35_‐treated group, CT‐NM/Res reduced mitochondrial ROS by about 63% and restored mitochondrial membrane potential, indicating that it effectively protected the neuronal mitochondria in vitro. As shown in Figure 5C (left), DiR‐loaded CT‐NM demonstrated highest ability of brain targeting compared to other groups. The results showed that the brain targeting ability of CT/NM NPs was 1.31‐ and 3.95‐fold higher than that of C‐NM and NM, respectively (Figure 5C, right side). Analysis of the mean escape latency and time spent in the Morris water maze showed that CT‐NM/Res restored the cognitive ability in AD mice.^[^ ^109^ ^]^

Considering the safety, multi‐functional use, and excellent biodegradablity, polymeric NPs can be used for targeting mitochondria in the treatment of NDs.

### 3.2 Inorganic NPs

Inorganic NPs not only have a large surface area, controllable size, and morphology, but also have good applicability for drug targeting and sustained release. Inorganic NPs, such as QDs, gold nanoclusters (AuNCs), and CeO_2_ NPs, are used for the diagnosis and treatment of NDs.^[^ ^110^ ^]^

#### 3.2.1 QDs NPs

**FIGURE 6:**
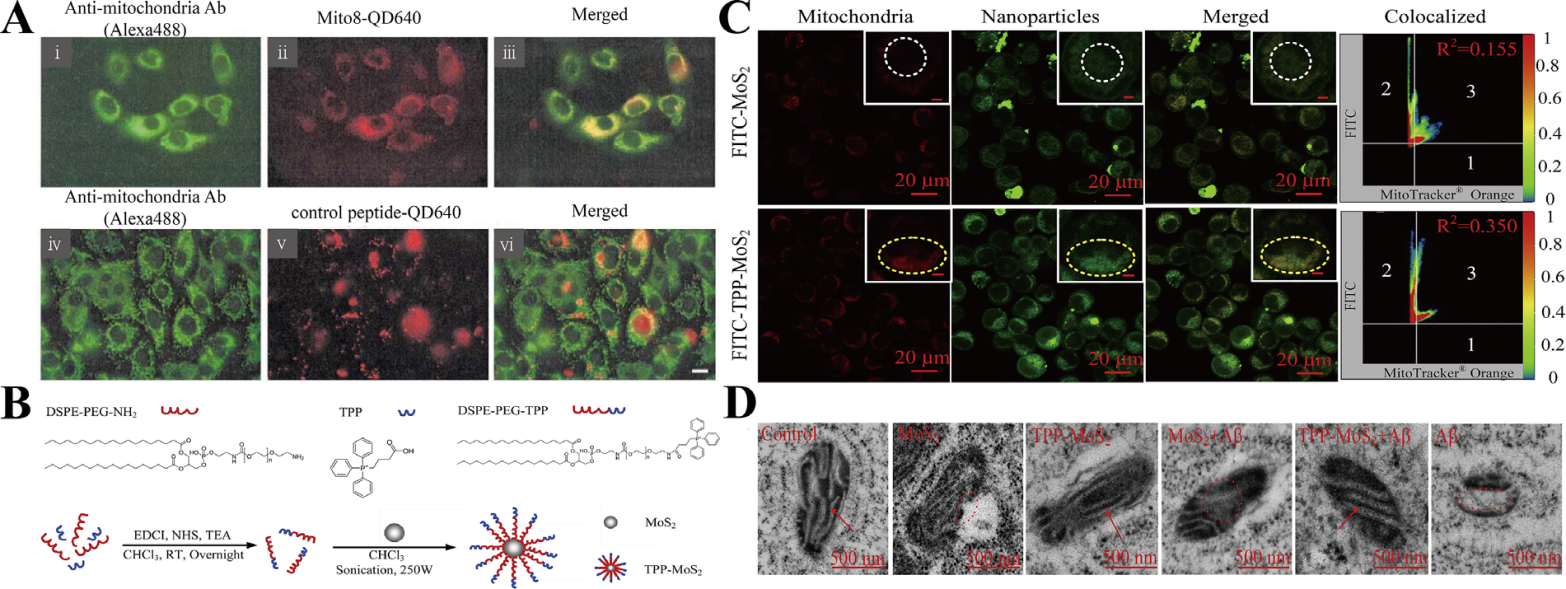
(A) Co‐localization level of Mito‐8 QD (i–iii) or control group (iv–vi) (red) with mitochondria (green) (scale bar = 10 μm). Reproduced with permission.^[^ ^115^ ^]^ Copyright © Owned by Center for Academic Publications Japan (Publisher). (B) Synthetic process of TPP‐MoS_2_ QDs. (C) Co‐localization images of mitochondria (red) with FITC‐MoS_2_ NPs/TPP‐MoS_2_ NPs (green). R^2^ represents the colocalization coefficient of green and red fluorescence. The color bar 0 (blue) to 1 (red) indicates the fluorescence intensity. (D) TEM images of mitochondria in different groups. Red arrows: complete mitochondrial cristae; red dashed circles: damaged mitochondrial cristae (scale bar = 500 nm). Reproduced with permission.^[^ ^112^ ^]^ Copyright © 2019 Elsevier Ltd. All rights reserved

QDs are a class of highly stable nanoscale semiconductors that have high quantum yield, high absorbency, narrow luminous spectrum, and high photobleaching resistance.^[^ ^111^ ^]^ Since the colors are excited at specific wavelengths, QDs can be used for the diagnosis and treatment of NDs.^[^ ^112^, ^113^, ^114^ ^]^ Hoshino et al. prepared mitochondria‐targeted NPs (Mito‐8‐QD), which can emit red fluorescence. Mito‐8‐QD (red) (Figure 6A, i–iii), but not control NPs (modified with non‐mitochondria‐targeted peptide) (Figure 6A, iv–vi), show higher co‐localization with mitochondria (green). Mito‐8‐QD acts as bio‐nanomachines for the diagnosis and treatment of mitochondrial dysfunction in NDs.^[^ ^115^ ^]^ Additionally, a TPP‐conjugated molybdenum disulfide QD (TPP‐MoS_2_ QD) was designed to penetrate the BBB and target the mitochondria (Figure 6B). As shown in Figure 6C, the co‐localization level of MoS_2_ QDs (green) with mitochondria (red) was significantly lower than that of TPP‐MoS_2_ QDs (*R* ^2 ^= 0.115 ± 0.01 and *R* ^2 ^= 0.350 ± 0.04, respectively).^[^ ^116^ ^]^ Notably, TPP‐MOS_2_ QD effectively prevented the disappearance of OMM and mitochondrial cristae caused by Aβ (Figure 6D), and decreased Aβ‐induced ROS level by 79.22% in BV‐2 cells compared to other groups. Interestingly, AD mice hippocampus showed less neuronal nuclei‐positive cells in control group than in TPP‐MOS_2_ QD group. TPP‐MOS_2_ QD also alleviated Aβ aggregate‐mediated neurotoxicity by reducing the inflammatory phenotype of microglia in vivo.^[^ ^112^ ^]^ Therefore, mitochondria‐targeted QD NPs are a promising strategy for theranostics of NDs.

#### 3.2.2 Gold NPs (AuNPs)

AuNPs have excellent light stability and can easily penetrate the BBB because of their ultra‐small size (< 3 nm) and biocompatibility.^[^ ^117^, ^118^ ^]^ In view of their antioxidant and anti‐inflammatory properties, AuNPs are used to treat NDs.^[^ ^119^ ^]^ Chiang et al. reported that AuNPs relieved Aβ‐induced mitochondrial dysfunction in cells via downregulating activities of caspase 3/9. Moreover, indicators related to mitochondrial mass or function (e.g., cytochrome C oxidase [COX] activity and mitochondrial membrane potential) and mitochondrial morphology were normalized in the AuNP group compared to control group.^[^ ^120^ ^]^ Similarly, AuNPs restored the antioxidant levels (catalase, SOD, and glutathione levels) in vivo, and maintained normal brain mitochondrial function by restoring ATP synthase activity in AD rat model.^[^ ^119^ ^]^

Additionally, functionalized AuNPs are used for the treatment of NDs.^[^ ^121^ ^]^ For instance, dihydrolipoic acid (DHLA)‐functionalized gold nanocluster AuNCs (DHLA‐AuNCs) attenuate neuroinflammation of the BV‐2 cell line. As ROS overproduction induced mitochondrial dysfunction and triggered neuroinflammation, ROS levels in different groups were determined. DHLA‐AuNCs significantly down regulated the percentage of ROS‐positive cells (41.3%) compared to the control group (86.5%). DHLA‐AuNCs eliminated the damaged mitochondria by inducing autophagosomes formation in the BV‐2 cell line.^[^ ^122^ ^]^ Moreover, Kalopanacis Cortex extract‐capped gold NPs (KC‐GNs) reduced intracellular ROS level by 60% compared to that of the control group, and attenuated mitochondrial membrane depolarization by about 35%.^[^ ^123^ ^]^ In addition to their therapeutic effects, DHLA‐AuNCs are also used to monitor Aβ (1–42) fibrillation process in real time.^[^ ^124^ ^]^

Therefore, AuNPs are promising strategies for targeting mitochondria and relieving oxidative stress and neuroinflammation in the treatment of NDs.

#### 3.2.3 Ceria (CeO_2_) NPs

**FIGURE 7:**
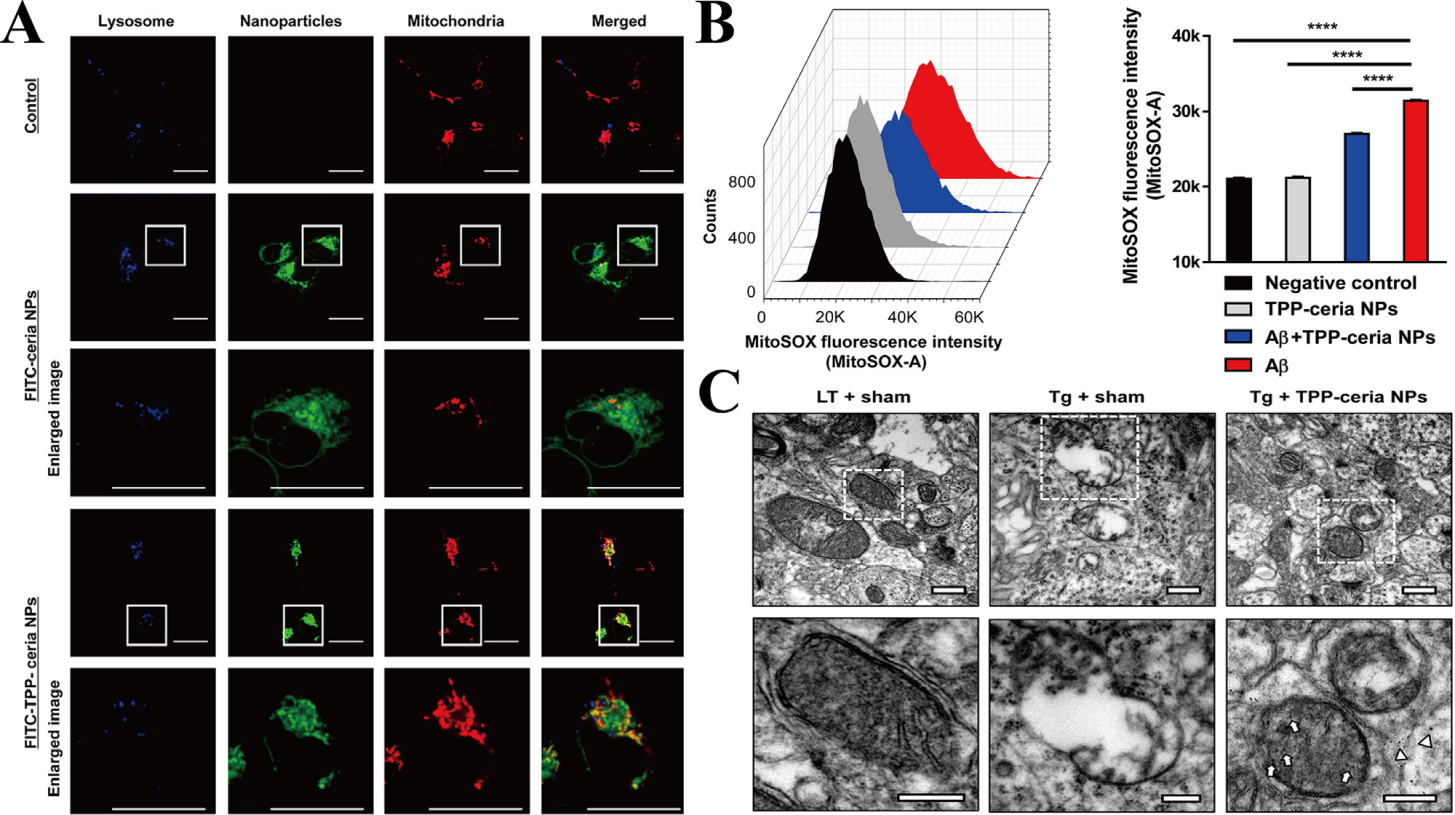
(A) Co‐localization images of mitochondria (red) with FITC‐ceria NPs/TPP‐ceria NPs (green) and lysosome (blue) (scale bar = 20 μm). Enlarged box area are shown below each image. (B) The representative images and statistic data of MitoSOX intensity in cell line. ****p < 0.0001. (C) TEM images of mitochondria morphologies (scale bar = 500 nm). Enlarged box area is shown below each image. Arrows: NPs in mitochondrial matrix; arrowheads: NPs in mitochondrial cytosol (scale bar = 250 nm). LT: littermate mice; Tg: 5 × FAD mice. Reproduced with permission.^[^ ^32^ ^]^ Copyright © 2016 American Chemical Society

By switching oxidation states between Ce^3+^ and Ce^4+^, CeO_2_ NPs perform their action as ROS scavengers. They cause oxygen defects in NP lattice structure and generate a cage for redox reactions.^[^ ^125^ ^]^ Hence, CeO_2_ NPs can imitate the catalytic activities of antioxidant enzymes to prevent neurodegeneration in NDs. Notably, CeO_2_ NPs accumulate on OMM or plasma membrane in rat cortical neurons. CeO_2_ NPs prevented Aβ‐induced mitochondrial fragmentation and decreased neuronal cell death by reducing dynamin‐related protein 1 serine 616 (DRP1 S616) hyperphosphorylation.^[^ ^126^ ^]^ Moreover, TPP‐conjugated CeO_2_ NPs (TPP‐ceria NPs) (green) co‐localized with mitochondria (red) in the cell line, whereas the ceria NPs were randomly distributed (Figure 7A). Additionally, the fluorescence intensity of MitoSOX (a fluorescent probe that can identify mitochondria‐specific ROS) indicated that TPP‐ceria NPs inhibit Aβ‐induced mitochondrial ROS in vitro (Figure 7B). TPP‐ceria NPs also mitigated the morphological mitochondrial damage by restoring shape of the disrupted cristae and vacuolar form (Figure 7C), and reduced the 4‐hydroxynonenal level (an oxidative stress marker) in vivo, suggesting a therapeutic effect of TPP‐ceria NPs in AD treatment.^[^ ^32^ ^]^

**FIGURE 8:**
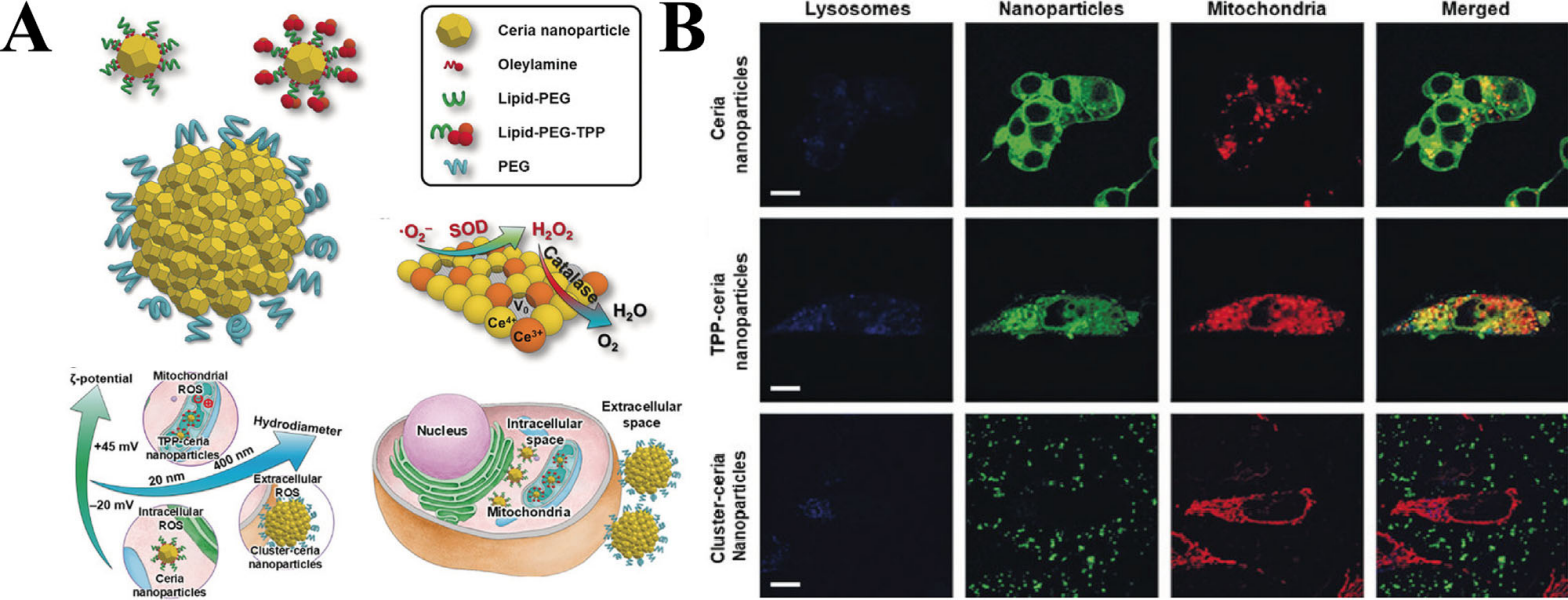
(A) Schematic illustration of the design and function of Cluster‐ceria NPs, TPP‐ceria NPs, and Ceria NPs. (B) Co‐localization images of different FITC‐NPs (green) with mitochondria (red) and lysosome (blue) (scale bar = 10 μm). Reproduced with permission.^[^ ^127^ ^]^ Copyright © 2018 Wiley‐VCH Verlag GmbH & Co. KGaA, Weinheim

Using ceria NPs, Kwon designed three types of NPs: cluster‐ceria, TPP‐ceria, and ceria NPs (Figure 8A). Among them, FITC‐TPP‐ceria NPs (green) group showed the best co‐localization level with mitochondria (red) compared to other groups (Figure 8B). Tert‐butyl hydroperoxide (tBHP)/H_2_O_2_ is intracellular/extracellular oxidative stress inducer. Therefore, the intracellular, mitochondrial, and extracellular ROS scavenging abilities of different groups were tested by tBHP + CellROX, tBHP + MitoSOX, and H_2_O_2_ + CellROX, respectively. The results of the mean fluorescence showed that the abovementioned three NPs selectively and effectively removed extracellular, mitochondrial, and intracellular ROS, respectively. Additionally, ceria NPs and TPP‐ceria NPs inhibited dopaminergic neuronal degeneration in the brain of PD mouse model.^[^ ^127^ ^]^ Those results suggest that ceria NPs and TPP‐ceria NPs are potential therapeutic strategies for PD treatment.

#### 3.2.4 Pd hydride (PdH) NPs

**FIGURE 9:**
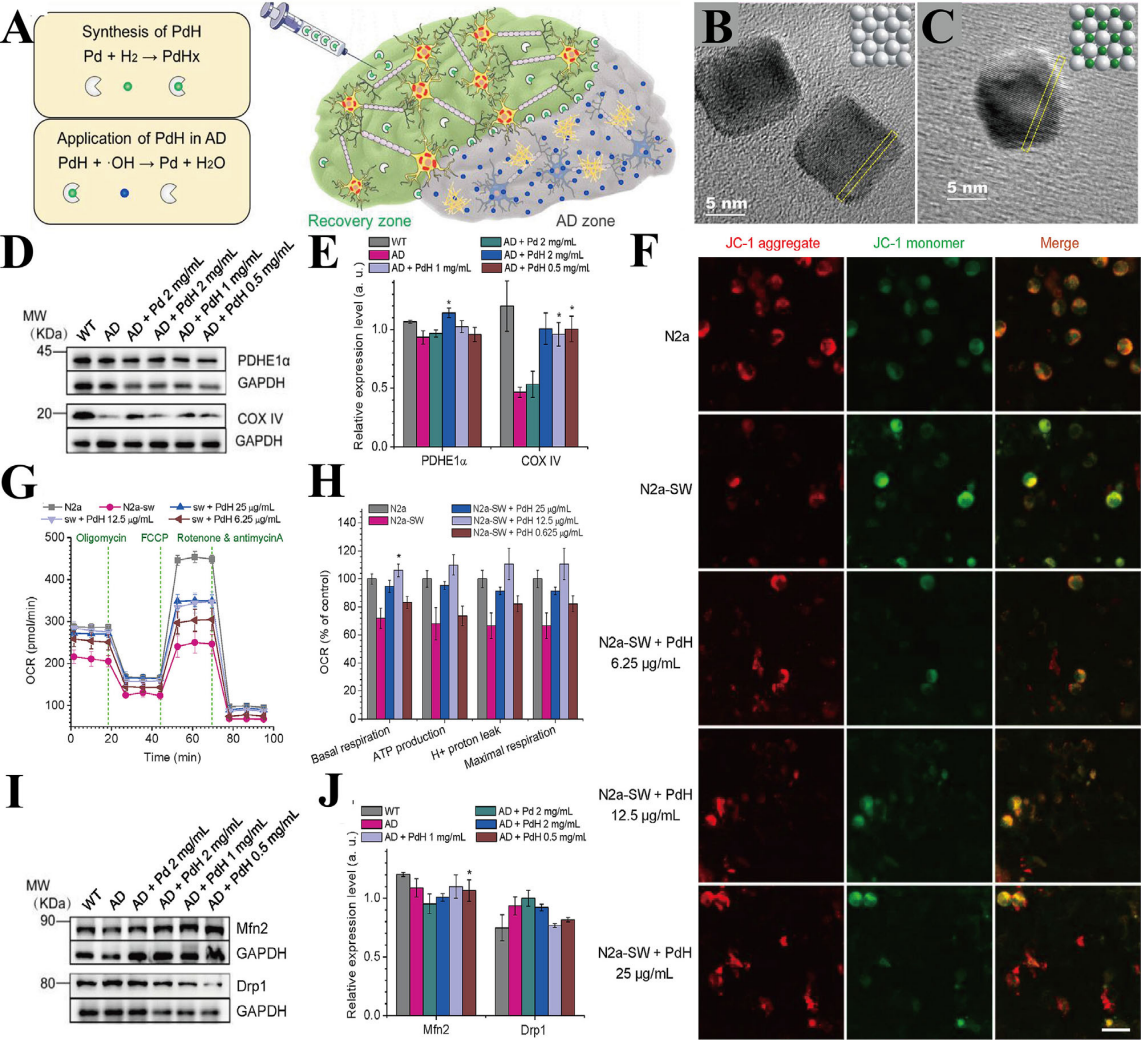
(A) Schematic illustration of synthesis and application of PdH NPs in AD treatment. (B,C) TEM images of PdH and Pd NPs. (D) Representative images and (E) statistic data of metabolism‐related proteins (PDHE1α and COX IV) expression in mice hippocampus. (F) Mitochondrial membrane potential of N2a (control cell line), N2a‐SW (AD model cell line), and N2a‐SW+different concentrations of PdH NPs measured by JC‐1 (scale bar = 20 μm). (G) Representative line graph and (H) statistic data of OCR (cell oxygen consumption rate), including ATP production, maximal respiration, H^+^ proton leak, and basal respiration. (I) Immunoblots pattern and (J) statistic data of mitochondrial fission/fusion proteins Mfn2/Drp1 expression in mice hippocampus. *p  <  0.05. Reproduced with permission.^[^ ^33^ ^]^ Copyright © 2019 Elsevier Ltd. All rights reserved

Hydrogen reduced the oxidative stress in AD by selectively removing the ROS.^[^ ^128^ ^]^ However, in view of their low solubility in biological systems, achieving in situ sustained release of hydrogen remains an obstacle. Zhang et al. developed Pd hydride (PdH) NPs as a hydrogen carrier to for sustained in situ release of bio‐reductive hydrogen to improve the cognitive ability of AD mice (Figure 9A). Transmission electron microscope (TEM) images showed that both Pd NPs and PdH NPs have a cubic shape and good dispersion ability (Figure 9B,C). Interestingly, compared with the control group, PdH NPs significantly increase the expression levels of mitochondrial damage marker, COX subunit IV (Figure 9D,E). Owing to the mitochondrial damage, N2a‐SW cells exhibited lower ΔΨm, specifically reflected as greater JC‐1 (a fluorescent probe for mitochondrial membrane potential detection) monomers (green) and less JC‐1 aggregates (red) compared to N2a cells. The ΔΨm of N2a‐SW cells was restored after treatment with different concentrations of PdH NPs (Figure 9F). The level of cell oxygen consumption rate (OCR), including ATP production, maximal respiration, H^+^ proton leak, and basal respiration (Figure 9G,H), was also restored to the normal level. Meanwhile, the expression levels of mitochondrial fission/fusion proteins Mfn2/Drp1 were restored in PdH NPs group compared to the other groups (Figure 9I,J). In addition, PdH NPs activated the Nrf2‐ARE pathway to protect neurons from oxidative damage.^[^ ^33^ ^]^

In summary, inorganic NPs are useful diagnostic and therapeutic strategies for NDs. By targeting neuronal mitochondria, inorganic NPs reduce oxidative stress and neuroinflammation in the CNS.

### 3.3 Biological membrane‐coated NPs

**FIGURE 10:**
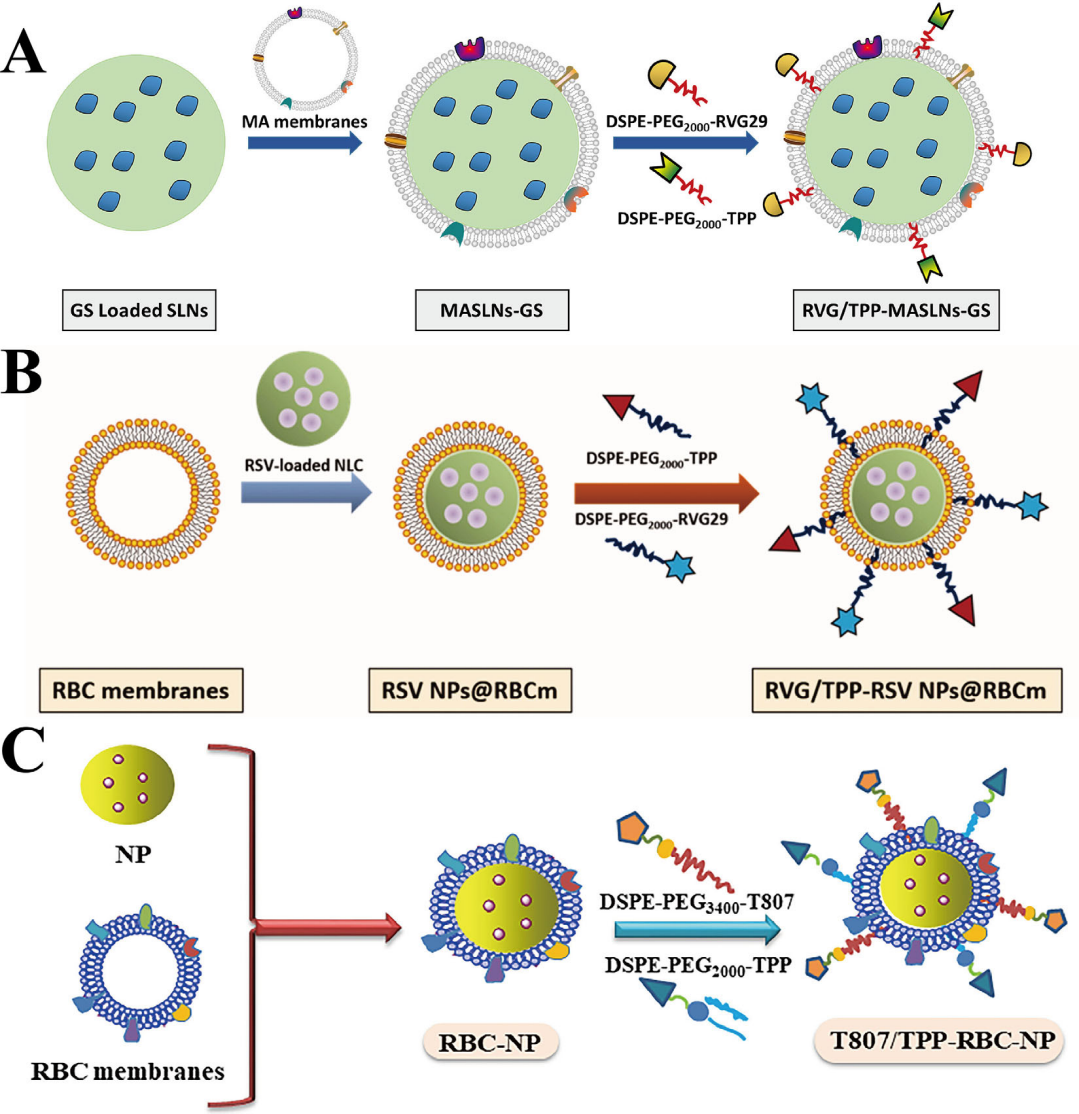
Schematic preparation of (A) RVG/TPP‐MASLNs‐GS, (B) RVG/TPP NPs@RBCm, and (C) T807/TPP‐RBC‐NP. Reproduced with permission.^[^ ^34^, ^35^, ^36^ ^]^ Copyright from 2020 The Authors. Publishing services by Elsevier B.V. on behalf of KeAi Communications Co., Ltd; 2020 The Author(s). Published by Informa UK Limited, trading as Taylor & Francis Group; 2020 Acta Materialia Inc. Published by Elsevier Ltd. All rights reserved

Biological membrane‐coated nanosystems have been widely studied because of their excellent biocompatibility and retention of unique cellular properties.^[^ ^129^ ^]^ Derived from white blood cells, macrophages, neutrophils, cancer cells, red blood cells (RBCs), and bacteria, the biological membrane coatings exhibit properties of the source cell.^[^ ^130^ ^]^ Hence, NPs take advantage of the multiple interactions between membranes and substrates, and provide an effective drug delivery platform for NDs. Several biomimetic delivery NPs (Figure 10A–C) for targeting mitochondria to treat NDs are described in the following paragraphs.

**FIGURE 11:**
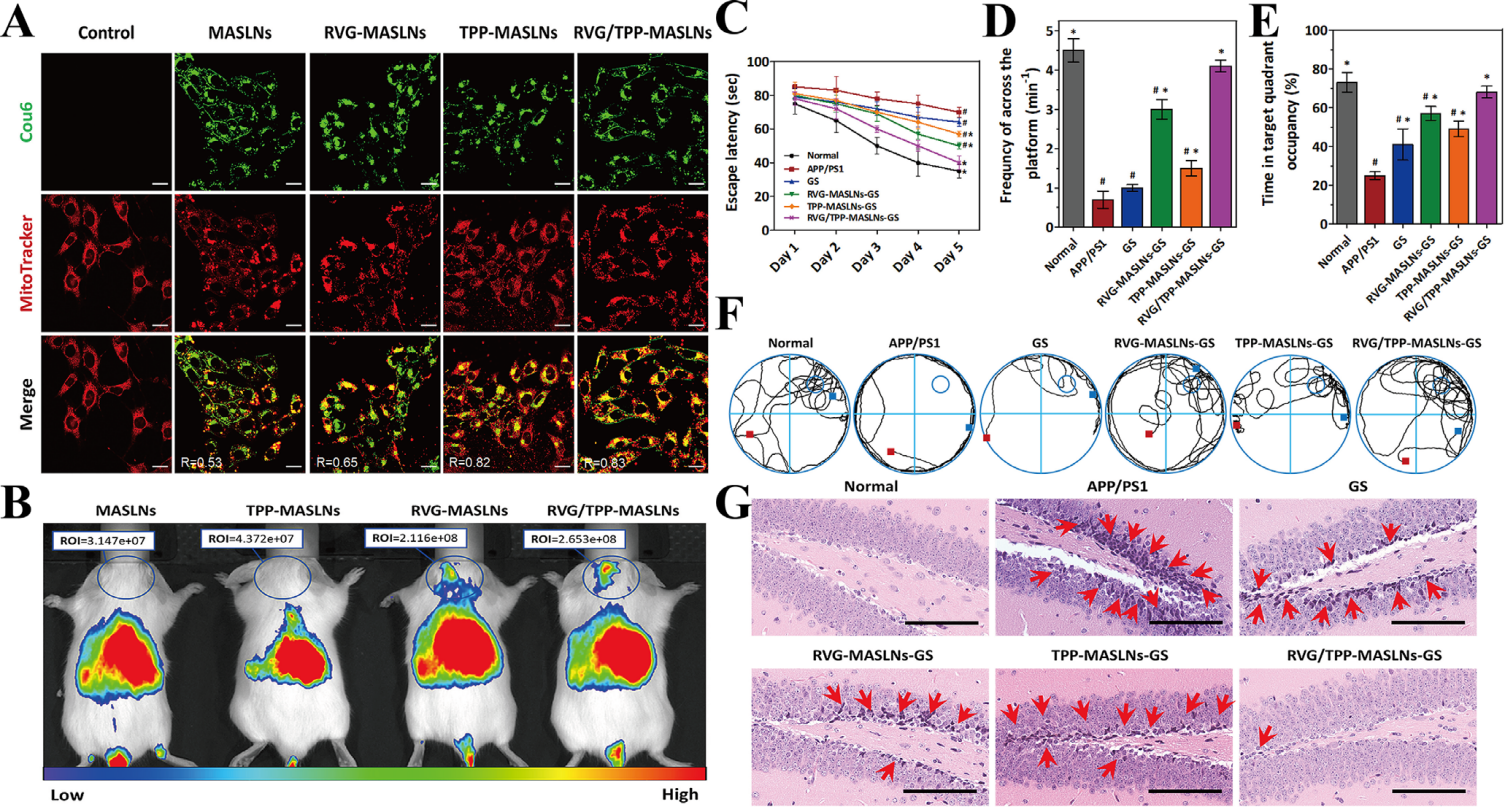
(A) Co‐localization imaging of various Cou6‐tagged NPs (green) with mitochondria (red) (Scale bars = 20 μm). (B) Distribution imaging of different DIR‐loaded NPs determined by IVIS Lumina II in vivo. (C) Escape latency time, (D) frequency across the area moved out of the platform, (E) time spent in the target quadrant, and (F) representative swimming paths of APP/PS1 mice in different NPs treatment group. (G) Images of AD mice hippocampal region in different treatment groups (scale bar = 100 μm). Damaged neurons are pointed by red arrows. Reproduced with permission.^[^ ^34^ ^]^ Copyright © 2020 The Authors. Publishing services by Elsevier B.V. on behalf of KeAi Communications Co., Ltd

Han et al. developed a macrophage (MA) membrane‐coated SLN and modified it with the mitochondria‐targeting molecule TPP and rabies virus glycoprotein (RVG29) to form RVG/TPP‐MASLNs, which showed positive effects on AD in vitro and in vivo. Due to the double‐mediated endocytosis, Cou6 labelled‐RVG/TPP‐MASLNs (green) preferably co‐localized with the mitochondria (red) of HT22 cells compared to the other groups (co‐localization coefficient *R* = 0.83), indicating their excellent neuronal mitochondrial targeting ability (Figure 11A). Furthermore, DIR‐tagged RVG/TPP‐MASLNs showed the highest ability in vivo to penetrate the BBB and reach the mice brain compared to other groups (Figure 11B). Notably, Genistein (GS)‐loaded RVG/TPP‐MASLNs (RVG/TPP‐MASLNs‐GS) is used for the treatment of AD. As an active natural flavonoid, GS prevents Aβ‐induced neuronal apoptosis in vitro.^[^ ^34^ ^]^ RVG/TPP‐MASLNs‐GS showed significant neuroprotective effects in Aβ‐damaged HT22 cells. Moreover, RVG/TPP‐MASLNs‐GS improved the cognitive deficits of APP/PS1 mice, including shortened escape latency (Figure 11C), increased frequency of crossing the platform (Figure 11D), prolonged time spent in the target quadrant after removing the platform (Figure 11E), and restored spatial learning ability (Figure 11F) compared to other groups. Interestingly, neuronal damage (pointed by red arrows) in the hippocampal region was reversed to varying degrees in different treatment groups. RVG/TPP‐MASLNs‐GS group demonstrated the best results compared to other groups, reflected by the orderly arranged and morphologically undamaged neurons (Figure 11G).^[^ ^131^ ^]^ In another study, the MA membrane was replaced into RBC membrane and mitochondria‐targeting RVG/TPP NPs@RBCm were synthesized (Figure 10B). When resveratrol (RSV) is encapsulated in RVG/TPP NPs@RBCm, they mitigate the mitochondrial oxidative stress to alleviate AD pathological features both in vitro and in vivo.^[^ ^35^ ^]^

RBC membrane‐camouflaged human serum albumin NPs (T807/TPP‐RBC‐NPs) are inserted into the DSPE‐PEG_3400_‐T807/DSPE‐PEG_2000_‐TPP molecules to deliver curcumin into neuronal mitochondria (Figure 10C). NPs loaded with curcumin (Cur‐T807/TPP‐RBC‐NPs) enhance the ability of curcumin to cross the BBB and target mitochondria, thereby relieving AD symptoms by suppressing neuronal death and mitigating oxidative stress in the mitochondria of the HT22 cell line and AD mice. Compared to the control group, the levels of oxidative stress biomarkers, including SOD, γ‐GT, MDA, and H_2_O_2,_ were restored to the normal range in the Cur‐T807/TPP‐RBC‐NPs group compared to other groups both in vitro and in vivo.^[^ ^36^ ^]^

Therefore, biological membrane‐coated NPs not only have suitable physicochemical properties, but also have unique biological functions (e.g., extended system retention time, reduced immune recognition, and reticuloendothelial system [RES] uptake), which may be helpful for ND treatment.

## 4 CONCLUSIONS AND FUTURE PERSPECTIVES

Mitochondrial dysfunction contributes to the progression of various NDs. Therefore, effective mitochondrial targeting and pathological recovery are important for successful treatment of NDs. Nanotechnology‐based mitochondrial‐targeted delivery may be an effective approach for the treatment of NDs.

In the previous sections, we described several physiological barriers (e.g., BBB, cell membrane, and mitochondrial membrane) that prevent NPs from targeting neuronal mitochondria. We also introduced different types of NPs designed according to the characteristics of the barriers. For instance, liposomes and polymer NPs are synthesized keeping in view that the mitochondrial membranes are rich in cardiolipin; cationic molecules, such as DQA and TPP, are synthesized based on the strong negative potential (ΔΨm) of the IMM; BBB‐penetrating and neuron‐targeting peptides, such as C3 and RVG29, are modified on the surface of NPs to enhance their BBB penetration and neuronal targeting ability. Importantly, several NPs are modified to form a dual targeting system for targeting neurons and mitochondria concurrently. Additionally, we discussed the role of different types of NPs in the ND models. Some NPs have natural antioxidant/anti‐inflammatory properties (e.g., AuNPs, CeO2 NPs, and PdH NPs), whereas some NPs have their own fluorescence (e.g., QDs NPs), which could be used for theranostics of NDs. Other NPs merely function as carriers (e.g., liposome‐based NPs, polymeric NPs, and biological membrane‐coated NPs) to encapsulate therapeutic drugs/fluorescein for treatment or imaging of NDs.

Although NPs are effective in the treatment of NDs, several limitations need to be addressed. First, central neurotoxicity of NPs needs to be further explored. Studies are needed to confirm the safety of NPs and to determine whether the neural uptake of NPs is due to receptor‐mediated endocytosis, pinocytosis, or axon transport mechanisms. Additionally, studies should also evaluate whether NPs can accumulate in a lesion without affecting the normal brain regions and whether they can affect the function of other organs by spreading through the circulatory system. Moreover, studies are needed to explore the safety and effectiveness of NPs in primate models, rather than mouse/rat models.

Second, delivery strategies of NPs into the mitochondria need further exploration. As most NPs targeted the mitochondria with almost the same modification molecules in this review (TPP and DQA), it is necessary to identify more biomarkers using the mitochondrial characteristics. New mitochondrial targeting molecules should be designed to improve the mitochondrial targeting efficiency and ND treatment outcomes. Moreover, intelligent analyses are required to ensure the drug accumulation and its physiological effects on mitochondria. In addition, physical properties of NPs, including size, shape, and charge, also affect the targeted delivery of NPs. The size of NPs affects their targeting effect, excretion pathways, systemic circulation time, and other properties. NPs with a diameter <5–6 nm easily cross the BBB and target the lesion, but are rapidly cleared by the kidneys. NPs with a diameter >100 nm are more likely to be modified by targeting molecules, but easily trigger immune responses, aggregate in the liver and spleen, and may be cleared by the RES.^[^ ^132^ ^]^ In general, it is necessary to determine the ideal size of NPs by considering the advantages and disadvantages of the abovementioned characteristics. The shape of NPs also determines their in vivo characteristics. Although spherical or near‐spherical NPs are most commonly used; they, in fact, provide limited binding sites for cell receptors. Non‐spherical NPs, such as those shaped as rods or sheets, effectively reduce RES clearance in vivo, prolong blood circulation time, and are more likely to accumulate at the target site.^[^ ^133^ ^]^ In conclusion, it is of great value to design NPs with an ideal shape for improving their therapeutic effect. Surface charge on NPs also impacts their targeting effect. Positively charged NPs (Zeta potential ξ < 10 mV) can induce serum protein aggregation. Negatively charged NPs (ξ > 10 mV) have a higher diffusion coefficient, but show strong RES absorption. Neutral NPs (±10 mV) have the lowest RES clearance and a longer systemic circulation time.^[^ ^134^ ^]^ Consequently, preparation of NPs with a modifiable charge may make it easier to target mitochondria.

Third, theranostic nanoplatforms require further investigation. Single imaging or treatment based on nanotechnology will not meet the current needs. Theranostic nanoplatforms simultaneously provide accurate real‐time diagnosis and treatment. Until now, few theranostics strategies exist in NDs treatment, such studies will lead to the application of theranostics to achieve the best clinical outcomes with the least cost and treatment time.

Finally, clinical trials are needed to confirm the effects of NPs in humans. Since studies barely probe the behavior of NPs on metabolism, in vivo studies are required to characterize the physical, chemical, and metabolic properties of NPs. Additionally, the mechanism underlying the ability of NPs to cross the physiological barriers in vivo need to be clarified to improve the current design of them. In addition, uniform criteria for determining the biodegradability of NPs in vivo need to be established. In summary, basic studies are required before nanotechnology can be used in routine clinical practice.

With further research in the above‐mentioned areas, a mature system based on nanotechnology may be designed for the clinical treatment of NDs.

## CONFLICT OF INTEREST

There are no conflicts of interest.
